# A universal multi-platform 3D printed bioreactor chamber for tendon tissue engineering

**DOI:** 10.1177/2041731420942462

**Published:** 2020-09-01

**Authors:** Adam J Janvier, Elizabeth Canty-Laird, James R Henstock

**Affiliations:** Institute of Ageing and Chronic Disease, University of Liverpool, Liverpool, UK

**Keywords:** Bioreactor, 3D printing, tendon, MSC, hydrogel

## Abstract

A range of bioreactors use linear actuators to apply tensile forces *in vitro*, but differences in their culture environments can limit a direct comparison between studies. The widespread availability of 3D printing now provides an opportunity to develop a ‘universal’ bioreactor chamber that, with minimal exterior editing can be coupled to a wide range of commonly used linear actuator platforms, for example, the EBERS-TC3 and CellScale MCT6, resulting in a greater comparability between results and consistent testing of potential therapeutics. We designed a bioreactor chamber with six independent wells that was 3D printed in polylactic acid using an Ultimaker 2+ and waterproofed using a commercially available coating (XTC-3D), an oxirane resin. The cell culture wells were further coated with Sylgard-184 polydimethylsiloxane (PDMS) to produce a low-adhesion well surface. With appropriate coating and washing steps, all materials were shown to be non-cytotoxic by lactate dehydrogenase assay, and the bioreactor was waterproof, sterilisable and reusable. Tissue-engineered tendons were generated from human mesenchymal stem cells in a fibrin hydrogel and responded to 5% cyclic strain (0.5 Hz, 5 h/day, 21 days) in the bioreactor by increased production of collagen-Iα1 and decreased production of collagen-IIIα1. Calcification of the extracellular matrix was observed in unstretched tendon controls indicating abnormal differentiation, while tendons cultured under cyclic strain did not calcify and exhibited a tenogenic phenotype. The ease of manufacturing this bioreactor chamber enables researchers to quickly and cheaply reproduce this culture environment for use with many existing bioreactor actuator platforms by downloading the editable CAD files from a public database and following the manufacturing steps we describe.

## Introduction

3D printing is a recently established technology for rapid prototyping and manufacturing – virtually all research institutions now have access to a fused filament fabrication (FFF) printer. 3D printing has had a transformative effect on researchers’ ability to rapidly prototype new designs and take a much greater level of control over experimental conditions, with these effects now being applied in the field of bioengineering. The free sharing of designs, tools and technologies has also enabled greater reproducibility of experimental approaches and methodology within the research community via online sharing platforms such as *Thingiverse* and *GradCAD*.^1^ Additive manufacturing can also be used to resolve long-standing challenges of reproducibility, comparability of data and lack of access to specific apparatus. In this investigation, we used 3D printing to overcome some of these challenges by designing an optimised bioreactor chamber for tendon tissue engineering with six independent wells. The bioreactor is designed for growing tissues under dynamic tensile loads and directly connects with many existing base platforms.

One of the principal applications for tensile bioreactors is for research into bioengineered tissues which experience strain in the body, for example, tendon. The predominant cell type in tendon is the tenocyte, and tenocyte-like cells can be generated from mesenchymal stem cells (MSCs) – a mechanosensitive cell capable of detecting changes in mechanical strain in their extracellular matrix (ECM).^2^ In vitro modelling of tendon development and healing has previously been studied using fibrin hydrogels by ourselves^3^ and other groups.^4–6^ Fibrin is a useful scaffold material for supporting tendon neosynthesis from MSCs since it provides adhesion motifs to enable cells to initially adhere before cell-directed remodelling, degradation and replacement with functional and tissue-specific ECM. During this phase, the fibrin network is contracted by the cells, forming a rod-like tendon structure ideal for tendon tissue engineering.^5^ Fibrin has also been shown to promote tenogenic gene expression patterns in MSCs and facilitate greater cell-generated collagen alignment when compared to hydrogels made from other materials, for example, collagen type I.^7^

MSCs have been differentiated into tenocyte-like cells under mechanical strain using an array of different methods.^8,9^ Consistent data have shown that dynamic loads in the range of 5–15% strain stimulate MSCs to undergo tenogenic differentiation, characterised by an increase in collagen type I production and an ECM with increased alignment and functional tensile strength.^10–12^ A wide range of strain rates and loading conditions are reported in the literature, from 1 to 15% strain, 0.1 to 1 Hz cycling frequency and 1 to 21 days culture across a multitude of species, cell sources and biomaterial scaffolds (Table 1). Finding a consistent methodology to inform new studies among this body of the literature presents a major challenge, and directly comparable data are scarce. Furthermore, many research groups are now using combination strategies for tissue engineering, using increasingly complex combinations of scaffolds, cells and biomolecules within bioreactors.

**Table 1.** Published work on cyclic tensile stimulation for tendon tissue engineering, showing the range in loading platforms and conditions.

	Strain	Duration	Frequency	Overall duration	Cell type	Hydrogel	Sample number per chamber	Bioreactor
Garvin et al.^13^	1%	1 h	1 Hz	8 days	Avian tendon internal fibroblasts	Collagen type I	n = 6, isolated culture wells	Flexcell
Nöth et al.^10^	12%	8 h	1 Hz	14 days	Human MSCs	Collagen type I	n = 1	Custom
Webb et al.^14^	10%	8 h	0.25 Hz	7 days	Human fibroblasts	Polyurethane	n = 4, shared culture well	Custom
Juncosa-Melvin et al.^15^	2.4%	8 h	0.2 Hz	12 days	Rabbit MSCs	Collagen type I	n = 5, isolated culture wells	Custom
Joshi and Webb^16^	5 or 12.5%	1–24 h	0.1–1 Hz	7 days	Human dermal fibroblasts	Tecoflex	n = 4, shared wells	Custom
Zhang and Wang^17^	4 or 8%	12 h	0.5 Hz	3 days	Rabbit tendon progenitor cell	Silicone membrane	n = 6, isolated culture wells	Custom
Barber et al.^11^	10%	2 h	1 Hz	10 days	Human MSCs	Electrospun polylactic acid	n = 4, isolated culture wells	Bose Electroforce Biodynamic 5200
Morita, et al.^12^	5, 10 or 15%	24 or 48 h	1 Hz	1–2 days	Human MSCs	Silicon monolayer	n = 5, isolated culture wells	Custom
Breidenbach et al.^7^	2.4%	5 h	1 Hz	14 days	Murine tendon progenitor cells	Fibrin and Collagen type I	n = 5, isolated culture wells	Custom
Heher et al.^18^	10% then 3%	6 then 8 h	Static	6 days	C2C12	Fibrin	n = 6, isolated culture wells	MagneTissue
Burk et al.^19^	2%	4, 8 or 24 h	1 Hz	1 day	Equine MSCs	Decellurised tendon	n = 1	Custom
Qiu et al.^20^	5%	12 h	1 Hz	14 days	Human MSCs	NDGA-crosslinked collagen fibre	n = 12, isolated culture wells	Custom
Youngstrom et al.^21^	3%	1 h	0.33 Hz	10 days	Tendon, bone marrow and adipogenic MSCs	Decellurised tendon	n = 1	Custom
Carroll et al.^22^	5 or 10%	4 h	0.5/1 Hz	21 days	Porcine MSCs	Fibrin	n = 6, shared culture well	Custom
Grier et al.^23^	10%	40 min	1 Hz	6 days	Human MSCs	Collagen -GAG	n = 24, isolated culture wells	Custom
Subramanian et al.^24^	2, 4 or 6%	2 h	0.1/1 Hz	7 days	Human adipose derived MSCs	Collagen type I	n = 4, isolated culture wells	Custom
Wu et al.^25^	4%	2 h	0.5 Hz	12 day	Human adipose derived MSCs	PCL nanofibrous woven scaffold	n = 6, shared culture well	CellScale MechanoCulture T6
Brandt et al.^26^	2%	1 h	1 Hz	3 days	Human adipose derived MSCs	Decellurised tendon	n = 3, shared culture well	Custom
Engebretson et al.^27^	2%	1 h	1 Hz	3 or 7 days	Rat MSCs	decellularized human umbilical vein	n = 4, isolated culture wells	Custom
Garcia et al.^28^	4%	2 h	1 Hz	14 days	Rat MSCs	PCL/HA electrospun scaffold	n = 6, shared culture well	CellScale MechanoCulture T6
Lee et al.^29^	10%	N/A	1 Hz	1, 3 or 7 days	Human MSCs	Decellurised tendon	n = 2, isolated culture wells	Custom
Liu et al.^30^	3%	12 h	0.2 Hz	7 days	Canine MSCs	Decellurised tendon	n = 6, shared culture well	Custom
Patel et al.^31^	5%	24 h	1 Hz	1 day	Bovine tenocytes	Poly(ethylene glycol) dimethacrylate	N/A	Custom
Raimondi et al.^32^	10%	12 h	0.5, 1 or 2 Hz	7 or 14 days	Porcine tenocytes	Collagen type I	n = 4, shared culture well	Custom
Ravelling et al.^33^	10%	12 h	0.05 Hz	3 days	Murine MSCs	Collagen type I	n = 1	Custom
Sensini et al.^34^	5%	1 h	1 Hz	7 days	Hs27	PLLA/ColI electrospun	n = 1	CellScale MCB1
Wunderli et al.^35^	1%	8 h	1 Hz	6 days	Murine tenocytes	Murine tendon fascicle	n = 8, shared culture well	Custom
Grier et al.^36^	5%	1 h	1 Hz	6 days	Human MSCs	Collagen type I/GAG	n = 24, isolated culture wells	Custom
Hsiao et al.^37^	4 or 8%	8 h	0.5 Hz	1 day	Rat tendon derived cells	Monolayer	n = 12, isolated culture wells	Custom
Atkinson et al.^38^	10%	8 h	0.67 Hz	14 days	Equine tenocytes	Collagen type I	n = 10, shared culture well	Custom
Banik et al.^39^	3%	2 h	0.01 Hz	21 days	Human MSCs	poly-(ε-caprolactone)	shared culture well	Custom
Ciardulli et al.^40^	10%	4 h	1 Hz	11 days	Human MSCs	Hyaluronate/Poly-Lactic-Co-Glycolic Acid (PLGA)/Fibrin	n = 1	Custom
Deniz et al.^41^	3% and 6%	30 min (first 2 days) then 60 min (final 8 days)	0.33 Hz	10 days	Human tenocytes	Poly (glycerol-sebacate) sheet	n = 1	EBERS TC-3
Talò et al.^42^	3%	30 min, 1 or 2 h	0.33 Hz	7 days	Rabbit MSCs	Decellurised tendon	n = 6, isolated culture wells	Custom
Tohidnezhad et al.^43^	2.5%	6 h	1 Hz	1 or 2 days	Rat tenocytes	Rat tendon	n = 1	Custom

A fundamental obstacle to comparing this increasingly complex and diverse research in tendon bioengineering is the variety of bioreactors used by researchers, with very few groups using identical or comparable platforms. Researchers use either custom-made tensile bioreactors or commercially available systems such as the EBERS TC-3 and the CellScale MC series. Our search of literature published between January 2016 and April 2020 indicates that of 24 published studies on cyclic loading in tendon tissue engineering, three groups published data generated using a CellScale bioreactor^25,28,34^ and one group published using an EBERS TC-3 bioreactor,^41^ with the remaining 20 using custom-designed bioreactors^19–24,26,27,29–33,35–40,42,43^(summarised in Table 1). There is a clear requirement for greater reproducibility and consistency in the methodological approach to enable both comparative research and translation towards effective clinical therapies. We propose that openly shared designs for low-cost, highly adaptable, 3D printable bioreactor chambers that can connect to a wide range of actuator platforms can help meet that need. The benefits of this are twofold: first, in providing a universal platform for comparison and reproduction of experiments, and second to enable a new generation of early career and interdisciplinary researchers, particularly scientists from developing countries.

Regardless of the specific bioreactor (commercial brand or custom-built), all of these systems share similar design characteristics: a culture chamber enabling mechanical stimuli to be applied to the cells in a sterile environment, with the force applied by a linear actuator controlled by displacement software. To apply tensile forces to cells, the biological material must form an interface with the loading hardware via a direct friction grip or clamp, pinning a mature (usually *ex vivo*) tissue in place, or incorporation of the biological sample with a loading anchor during the formation of the tissue. In most tensile bioreactors, one end of the sample is typically held in a fixed position, while the second is attached to a linear actuator, permitting movement in just one axis.

Our aims in this investigation were to design and manufacture a bioreactor culture chamber that could be produced using a standard benchtop 3D printer (e.g. Ultimaker 2+) with polylactic acid (PLA) filament and a small number of easily sourced, commercially available parts. The bioreactor chamber was designed to be adaptable to a range of base actuator platforms (here, we have used the EBERS TC-3 and the CellScale MCT6). To maximise the versatility of the bioreactor and improve upon existing devices, we began by establishing several design criteria based on projected applications using information available in the literature. The first of these design criteria was that the bioreactor culture chamber should have six isolated wells to enable either simultaneous stretching of up to six differently treated samples, or an effective n = 6 number of experimental repeats. This design criterion was paramount since many commercially available bioreactor chambers have a single unsegregated volume which does not allow for statistically distinct repeats (see Table 1). This also overcomes a limitation imposed by the conventional single-well setup, which necessitates serial rather than parallel experimental runs and forces a compromise between experiment loading time and n-number. Based on the available literature, we determined that each of the six wells should have a displacement volume of 8 mL to allow the culture of a variety of engineered tissues, while ensuring the tissues can remain submerged in 3–5 mL culture media subject to the application, for example, the size of the engineered tissue construct. The media volume was carefully considered based on the volumes typically used in a six-well cell culture plate, and optimised to provide sufficient nutrient availability and buffering during culture, while minimising media wastage and maximising the concentration of secreted analytes. The armature design for many commercially available bioreactors permits a 0- to 25-mm experimental strain displacement range, and we used this as a benchmark limit for our bioreactor chamber with the added specification that the displacement be delivered equally to all wells. To ensure consistent loading in each well, we tested displacement up to a maximum 50% strain. To validate the biological effects of the bioreactor, we used an established strategy: MSC-tenocyte differentiation within a fibrin hydrogel, and performed full biocompatibility and sterility testing of the components.

## Materials and methods

### Bioreactor chamber

**Figure 1.**
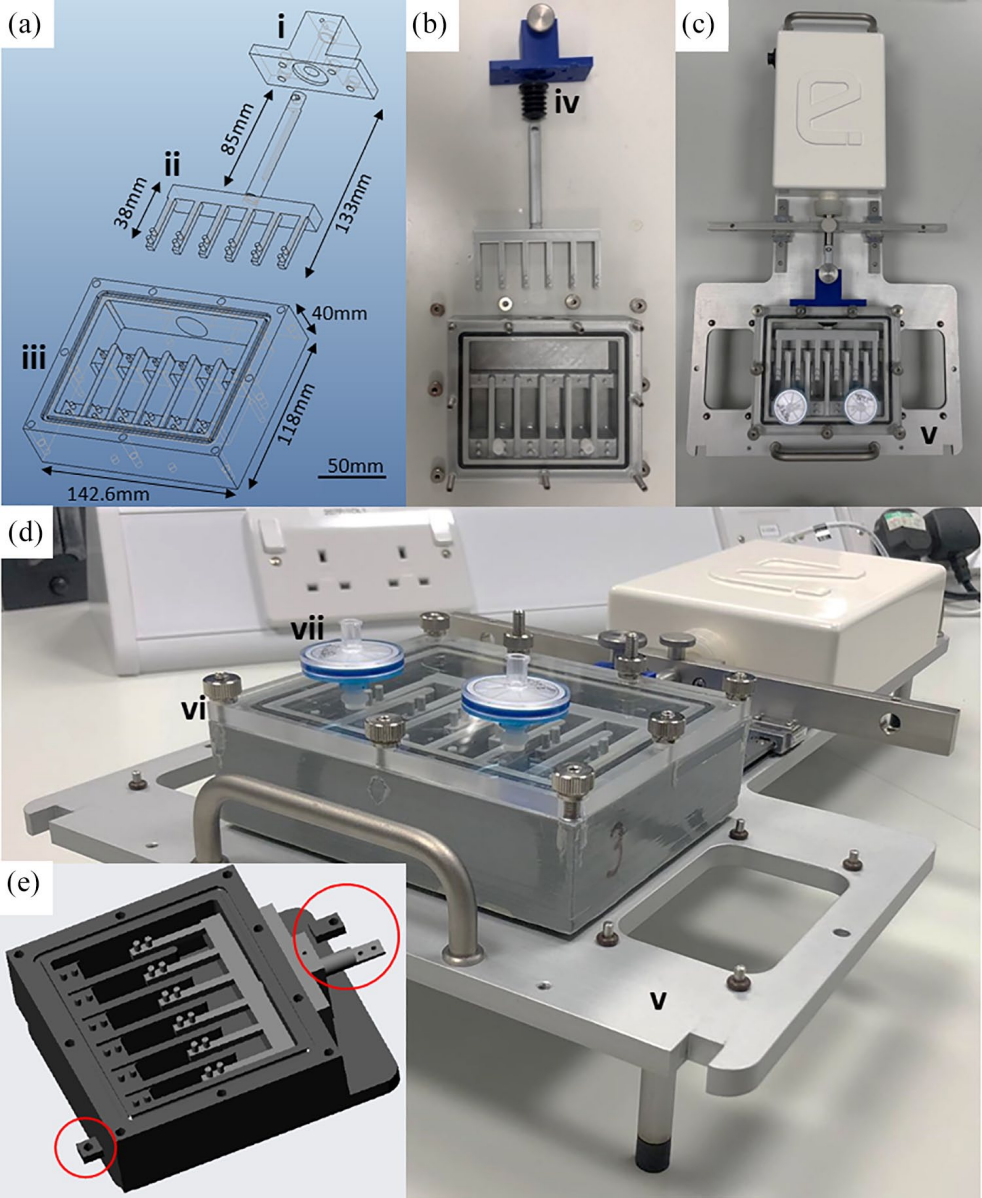
**3D printed culture chamber for tensile stimulation of 3D tissue-engineered tendon.** Wildfire 5 CAD software was used to design the culture chamber, shown in (a) as an exploded CAD drawing, highlighting (i) the tensile arm runner connecting to the EBERS TC-3 bioreactor, (ii) the tensile arm splitting the main drive shaft into the six-well format and (iii) the tissue culture chamber and base plate. These components of the culture chamber were 3D printed in PLA (b) and mounted onto an EBERS TC-3 baseplate (c and d), ensuring gas sterility with a rubber bellow (iv) and attached securely to the aluminium baseplate (v). The culture chamber lid (vi) was manufactured from the 6-mm clear polycarbonate sheet with drilled holes for 9 mm securing screws and two Luer lock fittings for 0.2 µm nylon button filters for sterile gas exchange (vii). Minor design modifications were required to mount the bioreactor onto the CellScale MCT6 (e): alternative bolt points were added and the base of the chamber body raised slightly to align with the actuator, and minor changes were made to the length and end attachment point of the tensile arm (circled).

3D printed components were designed using Pro/Engineer Wildfire 5, saved as STL (STereoLithography) files and printed with the Ultimaker 2 + FFF (Fused filament fabrication) 3D printer using PLA filament (3DGBIRE, UK). The main body of the 3D printed culture chamber was fully coated in XTC-3D ‘Smooth on’ high-performance 3D print coating (an oxirane epoxy resin used to waterproof the chamber), cured overnight and washed with PBS. The XTC-3D was prepared as specified by the manufacturer; part A (resin) and part B (hardener) were mixed at a ratio of 2:1 and applied as a thin coat to the base and walls of each culture well, the chamber was left to cure overnight. The base of each culture well was then coated with polydimethylsiloxane (PDMS) Sylgard-184 (Sigma-Aldrich, UK) to prevent the tissue-engineered tendon from sticking to the well base during loading. The Sylgard-184 was prepared according to the manufacturer’s instructions, and mixed with the curing reagent at a ratio of 9:1 in a 50-mL centrifuge tube, then left to mix on a rotormixer for 10 min at room temperature. One millilitre of the Sylgard-184 mixture was pipetted into the base of the culture well and a level coating was ensured by placing the chamber on a flat surface, then left to cure for 3 days at room temperature. Degassing was not required as bubbles were not observed in either the XTC-3D or the Sylgard-184. Once the XTC-3D and Sylgard-184 were fully cured and hardened the culture wells were washed through six changes of PBS to remove any residual cytotoxic monomers. The culture chamber was constructed as three 3D printed components: the main chamber body, the tensile arm and the tensile arm runner, plus a machine-cut transparent polycarbonate lid (Figure 1). Four 2.5-mm diameter holes printed on the front face of the chamber body were threaded (M3) to affix the tensile arm runner to the chamber body. The six-way tensile arm with bellow attached was secured in place by the tensile arm runner, allowing only forward and reverse motion, while the bellow created a flexible gas-tight seal. Nine 4.2-mm holes were printed on the top of the chamber body and were threaded with an M5 thread to secure the lid using M5 grub screws and thumb nuts. A 3-mm O-ring groove was printed into the upper face of the chamber body to accommodate a 134 mm × 3 mm Viton rubber O-ring, forming a gas-tight seal between the chamber and the polycarbonate lid. The transparent lid was cut from a 6-mm clear polycarbonate sheet (RS Components, UK), and 6-mm holes were drilled to align with the printed holes on the chamber body. Two holes for standard Luer lock-screws allowed the fitting of two replaceable 0.2-µm nylon syringe capsule filters (all Cole-Parmer, UK) for sterile air flow. Final assembly therefore required seven commercially available secondary components: Bellow, M3 screws, M5 grub screw, thumb nut, O-ring, Luer locks and 0.2-μm nylon syringe capsule filters.

### Tendon attachment frames

**Figure 2.**
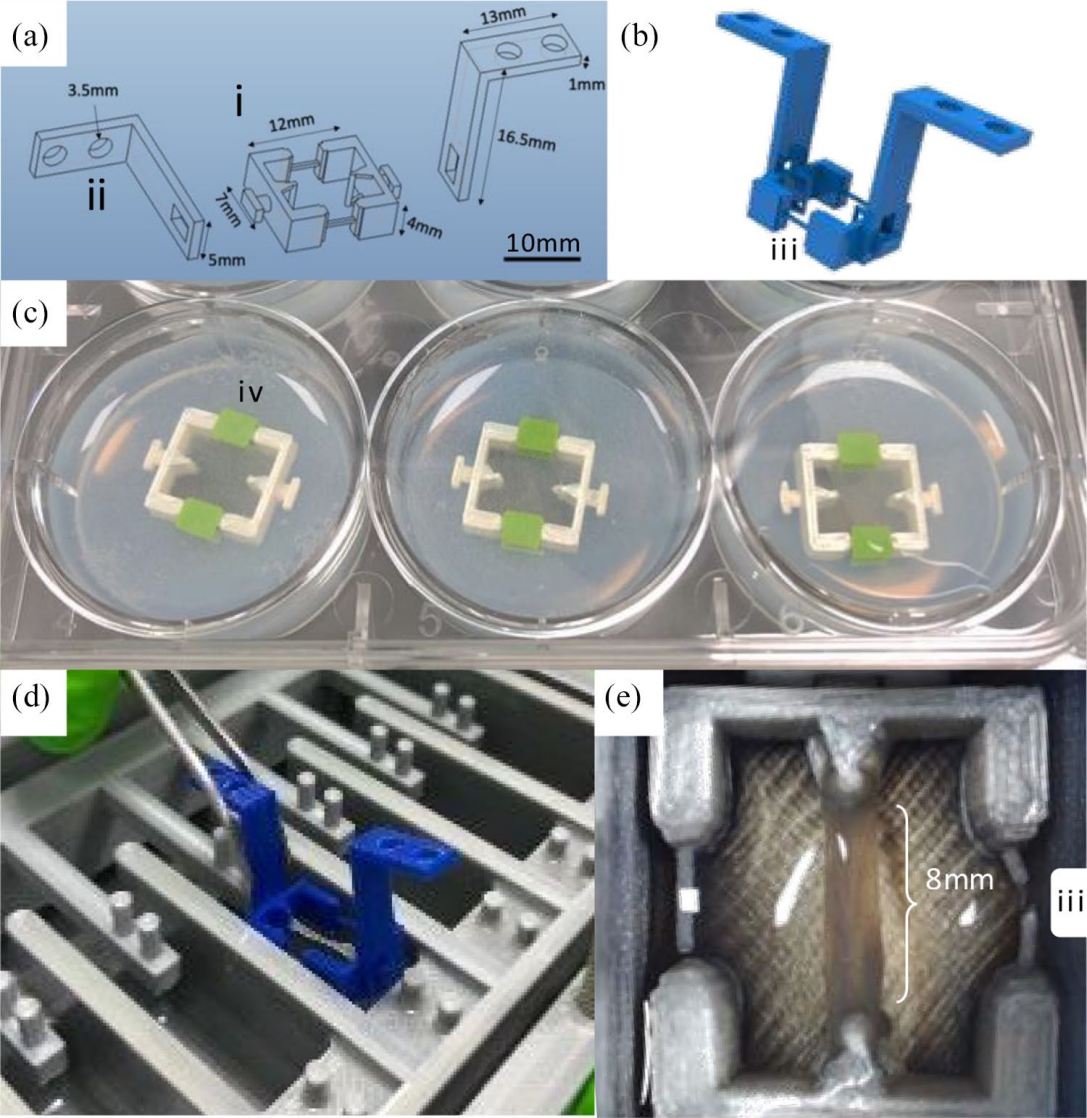
**Attachment of tissue-engineered tendon within the bioreactor.** Anchor frames were designed to attach to the six tensile arms and deliver stretching forces to fibrin hydrogels containing human MSCs. Wildfire 5 was used to design the anchor frame (a) comprising a tissue-engineered tendon attachment bracket (i) with separate adapter arms (ii) which attach securely to the frame through a 90° rotation of the arm (b). To enable the tendons to form at a constant length, two thin breakable spars connected both halves of the frame that were severed at the onset of loading (b, iii). These spars had removable 3D printed covers to provide an enclosed perimeter mould to separate the agarose from the cell-seeded fibrin hydrogel (c, iv) The two-part assembly enabled pre-culture of cells in fibrin hydrogels in standard well plates (c) before transfer to the loading chamber (d and e).

3D printed tendon attachment frames were designed to contain the fibrin tendon construct and integrate the attachment points for tensile loading, which were connected to the chamber main body (point A) and the six-way tensile arm (point B). The solid PLA anchor frames were not coated in the XTC-3D resin or Sylgard-184. For practical purposes, the tissue-engineered tendons were initially made in six-well plates, requiring a two-part assembly with removable connectors for points A and B which attached securely through a 90° rotation. Once prepared, the frames containing tissue-engineered tendons were moved into the bioreactor chamber and the connecting spars broken with sterile scissors. The distance between the tendon attachment points was 8 mm (12 mm between rear of attachment points), the frame width was 7 mm and depth was 4 mm containing a total volume of 330 μL (Figure 2(a)).

### Displacement validation

The 3D printed culture chamber was mounted onto the EBERS-TC3 base platform (frame, actuator and control software). The displacement of the printed tensile arm was measured using an HD USB camera (MicroDirect, Celstron), distances were calculated using ImageJ (Fiji) and analysed using GraphPad Prism 8.

### Cell culture

Human MSC (hMSCs) (Lonza) were cultured at 37°C in normoxia and 5% CO_2_. The medium used throughout the investigation contained DMEM (Gibco, Life Technologies), 10% foetal bovine serum (FBS) (Gibco, Life Technologies), 2% antibiotic-antimycotic (Sigma-Aldrich), 1% non-essential amino acids (Sigma-Aldrich) and 1% l-glutamine (Sigma-Aldrich).

### Biocompatibility

The lactate dehydrogenase (LDH) assay (Pierce, Thermo Scientific) was used to determine the biocompatibility of the materials which interfaced with the cell culture media – PLA, XTC-3D ‘smooth on’ oxirane epoxy resin, and PDMS Sylgard-184. Human MSCs (passage 3) were seeded in triplicate in wells of a 12-well plate at a density of 1.5 × 10^5^ cells/mL and allowed to attach for 12 h. Pieces of PLA (5 mm × 5 mm × 1 mm, ~5 g) were added in triplicate to experimental wells, while PLA coated with XTC-3D oxirane resin (5 mm × 5 m × 1 mm, ~5 g) was used to represent the walls of the culture wells. In this initial test, the XTC-3D oxirane resin was only washed once in PBS. The cells were incubated with the materials overnight and compared to unmodified control wells. The LDH activity within the media was quantified using the SPECTROstar Nano microplate reader (BMG LABTECH). The biocompatibility/cytotoxicity assay was then repeated using the fully coated, cured and washed bioreactor chamber, which included both the XTC-3D oxirane resin-coated walls and the Sylgard-184 coated well bases. The 3D printed culture chamber was sterilised with 70% ethanol; initially, all components were disassembled and washed in 70% ethanol before drying within a sterile cell culture hood for 1 h. Once dry the 3D printed culture chamber was assembled and washed in 70% ethanol again and left to dry in a flow hood, washed again three times with PBS and left to dry. Human MSCs were seeded onto cover slips at a density of 1.5 × 10^5^ cells/mL and placed in the bioreactor wells, then incubated overnight at 37°C and assayed for LDH activity as described above.

### Tissue-engineered tendon

The tendon attachment frames were sterilised in 70% ethanol, washed in sterile PBS before being fixed into position within a six-well plate by dispensing 2.5 mL sterile 4% agarose around the outside of the frame (Figure 2(b)). MSCs were dissociated from the tissue culture plastic at passage 3 using trypsin and seeded at 1.25 × 10^6^ cells/mL in fibrin to create individual tissue-engineered tendons from 75 μL (20 mg/mL) fibrinogen, 25 μL (200 Unit) thrombin (both Sigma-Aldrich) and 230 μL media containing the cell suspension. The tissue-engineered tendons were cultured in the six-well plate for 14 days with media changes every 48 h with the addition of 1 mg/mL 6-aminocaproic acid (Sigma-Aldrich) to inhibit fibrin degradation during the contraction phase.^44^ After 14 days, the tendon attachment frames were moved from the six-well plate and placed into the bioreactor chamber using the adapter arms. Once in the bioreactor chamber, the spars were broken using sterile scissors. The tissue-engineered tendons were cultured in 3.5 mL of media with 800 μM of freshly prepared L-ascorbic acid (Sigma-Aldrich) with media changes every 48 h. Mechanical loading was applied as 5% strain (based on the initial 8 mm length of the tissue-engineered tendon) for 5 h a day at 0.5 Hz for five consecutive days per week over 21 days.

### Histology

Two tissue-engineered tendons from both control and loaded groups were removed from the bioreactor chamber, washed three times in PBS and fixed in neutral buffered formalin (NBF) for 30 min at room temperature. Fixed samples were washed three times in PBS and stored in 0.01% sodium azide in PBS at 4°C, then mounted in paraffin wax and sectioned using a Leica RM2245 microtome, generating 6-μm slices. The sections were stained for haematoxylin and eosin (H&E), Picrosirius red and Alizarin red. H&E and Alizarin red images were acquired using a Nickon eclipse Ci, while Picrosirius red images were acquired using an Olympia BX60 in both brightfield and polarised light. All microscopy images were taken at ×10 objective.

### Protein analysis

Tissue-engineered tendons were removed from the bioreactor chamber (n = 3) and frozen at −80°C, thawed and ground using a motorised pestle (Sigma-Aldrich). Soluble protein was extracted in RIPA buffer (Sigma-Aldrich), and insoluble material was digested in 25 μg/mL pepsin (Sigma-Aldrich), 0.1 M acetic acid (Sigma-Aldrich) and 200 μg/mL EDTA (Sigma-Aldrich). Soluble and digested protein were combined and total protein concentration measured using the BCA assay. Extracted proteins were analysed using a standard dot blot procedure with 15 μg of total protein pipetted onto a nitrocellulose membrane (Protran, Sigma-Aldrich). Once the protein dot had dried, total protein was stained using Ponceau S and imaged using the ChemiDoc chemiluminescence detector (Bio-Rad). Ponceau S stain was removed with three times washes of TBS-T buffer, the membrane was then blocked for 1 h at room temperature with 5% reconstituted dehydrated milk in TBS-T. The membrane was then incubated overnight at 4°C with either anti-collagen Iα1 (1:1000 in blocking buffer) (ab138492, Abcam) or anti-collagen IIIα1 (1:1000 in blocking buffer) (ab7778, Abcam) primary antibodies. The primary antibody was removed and the membrane washed three times with TBS-T before incubating at room temperature for 1 h in the anti-rabbit secondary antibody (1:5000 in blocking buffer) (ab205718, Abcam). The membrane was washed a further three times with TBS-T. Protein was detected by staining the membrane with Western lightning enhanced chemiluminescence (ECL) Pro (PerkinElmer) before imaging using the ChemiDoc chemiluminescence detector (Bio-Rad). Densitometry was performed using ImageJ.

### Statistical analysis

Statistical analysis was measured with GraphPad Prism 8 using a Student’s t-test or one-way analysis of variance (ANOVA) with multiple comparison tests. Significance was set at *p* < 0.05. Data are presented as mean values ± standard deviation.

## Results

The bioreactor chamber was designed and manufactured using equipment available in most research intuitions including an FFF 3D printer, milling machine and pillar drill (Figure 1(a) and (b)). This version was developed for use with the EBERS TC3 platform, specifically through adapting the connections to the platform base plate (Figure 1(c) and (d)). The cross-platform adaptability of the bioreactor chamber was demonstrated by the minor design modifications required to mount it onto the CellScale MCT6 (Figure 1(e)). For the bioreactor chamber to be used with the CellScale MCT6, alternative bolt points were added to the base of the chamber body, and minor changes were made to the length and the end attachment point of the tensile arm. The rest of the chamber was unchanged between the EBERS-TC3 version and the CellScale MCT6.

The bioreactor was composed of four main components. Three of the four components were 3D printed (Figure 1(a)), the six-way split tensile arm, the tensile runner and the chamber body, while the transparent polycarbonate lid was manufactured by machining polycarbonate sheet. In case, transparency was not required, or the tools for cutting and drilling polycarbonate unavailable, CAD for an opaque lid was also created. Final assembly required seven additional commercially available components: Bellow, M3 screws, M5 grub screw, thumb nuts, O-ring, Luer locks and 0.2-μm nylon syringe capsule filters (Figure 1(b) and Table 2). These secondary components were for general assembly or to generate an air tight seal to ensure sterility. The chamber body took approximately 35 h to print with 175 g of material (22.13 m of PLA reel), while the tensile arm and runner took approximately 8 h to print with 40 g of material (5 m of PLA reel). Once printed, the screw threads were cut and components coated with XTC-3D High-Performance 3D print coating (Smooth-on) and Sylgard-184 to cover any micropores between fused filament layers and make it fully impermeable to culture media and the sterilisation solutions. Total assembly time from the onset of printing to a culture-ready bioreactor was 5–6 days, at a unit cost of £40–50.

**Table 2.** Full components list for the bioreactor chamber.

Name	Description	Supplier	Product code
Ultimaker 2+	FFF 3D printer	RS Components	918-8695
PLA filament	3D printing filament	RS Components	134-8190
Bellow	Flexible seal	Don Whitley Scientific	SP-90.007.006
Grub screw	M5 x 30 mm	Accu.co.uk	SSU-M5-30-A2
Polycarbonate sheet	1.25 m x 610 mm x 6 mm	RS components	681-665
O-ring	3 mm cross section, 134 mm circumference. VITON rubber	Simply bearings	simplybearings.co.uk
XTC-3D	Waterproof resin	Smooth-on	benam.co.uk/xtc-3d
Thumb nut	M5	RS components	664-4886
Screw	M3	RS components	280-981
Sylgard 184	Low friction seal	Farnell	101697
Luer lock adapter	Attaches air filter	Cole Parmer	OU-30800-00
0.2-µm syringe filter	Air filter	Cole Parmer	16534—————K
Thumb screw	M3	Accu.co.uk	SKT-M3-10-A1
M3 Thread insert	M3	Accu.co.uk	HSTI-M3-A2
M5 Thread insert	M5	Accu.co.uk	HSTI-M5-A2
CAD files	https://www.thingiverse.com/Janvier1/collections/tensile-stimulation-bioreactor		

FFF: fused filament fabrication; PLA: polylactic acid.

The chamber was designed to accept six individual 3D printed anchor frame assemblies containing tissue-engineered tendons, which were pre-made in six-well plates for practical and ergonomic reasons (Figure 2(c)). The design for the frames includes two internal tendon attachment points which avoid the use of grips or clamps to secure the ends of the tissue and were optimised to distribute the mechanical stress through the tissue without breaking the fibrin hydrogel scaffold.^38,45^ After 14 days culture in six-well plates, the cell-seeded fibrin hydrogels formed tendon-like tissues between the attachment points which were easily removed from the well plate and slotted into individual wells of the bioreactor chamber. At this stage, two 90° adapter arms were attached (Figure 2(b)) to connected points A and B of the frame to the corresponding points on the bioreactor chamber and six-way tensile arm (Figure 2(d)). The anchor frame included two thin, breakable spars designed to ensure each tissue-engineered tendon formed at a uniform length during the contraction phase and prevented stress on the developing tissue prior to installation within the bioreactor. Following the insertion of the anchor frame into the bioreactor chamber, the spars were cut with scissors (Figure 2(e)), allowing the tendon to be stretched. When in the six-well plate, the spars were covered with removable covers to prevent the fibrin from running out of the frame during gelation (Figure 2(c)). The six-way tensile arm had a maximum displacement of 18 mm, with the extension limit of the frame within each culture well being 8 mm, equating to an upper limit of 112.5% strain (>2x original sample length). The maximum media capacity of each well was designed to allow the culture of a variety of engineered tissues, while ensuring the tissues can remain submerged in 3–5 mL culture media subject to the application, for example, the size of the engineered tissue construct. The media volume was carefully considered based on the volumes typically used in a six-well cell culture plate, and optimised to provide sufficient nutrient availability and buffering during culture, while minimising media wastage and maximising the concentration of secreted analytes. For this particular study, 3.5 mL of culture media ensured the tissue-engineered tendon was fully submerged throughout culture and loading.

**Figure 3.**
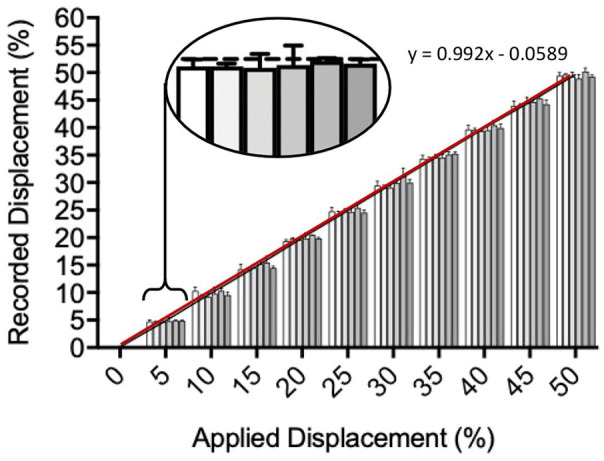
**3D printed culture chamber performance.** The linear displacement of the primary tensile arm by the software-controlled drive motor was shown to result in equal arm movement across each of the six wells of the printed chamber (n = 3 technical repeats per well and n = 6 experimental repeats across the chamber, significance measured using one-way ANOVA with Kruskal–Wallis multiple comparison tests).

The performance accuracy and calibration of the bioreactor were validated by measuring the displacement of the six-way tensile arm in each well when mounted onto the EBERS TC-3 bioreactor (Figure 3). The displacement of the six individual tensile arm end points was recorded as the percentage displacement of the tissue-engineered tendon within the 8 mm attachment frames. At 4 mm (50% strain), the displacement across all wells was within 0.6% of the programmed value, with no significant differences between wells at strain rates 5–50% across the wells (*p* > 0.09) and a linear correlation between programmed and observed values (R^2^ = 1).

**Figure 4.**
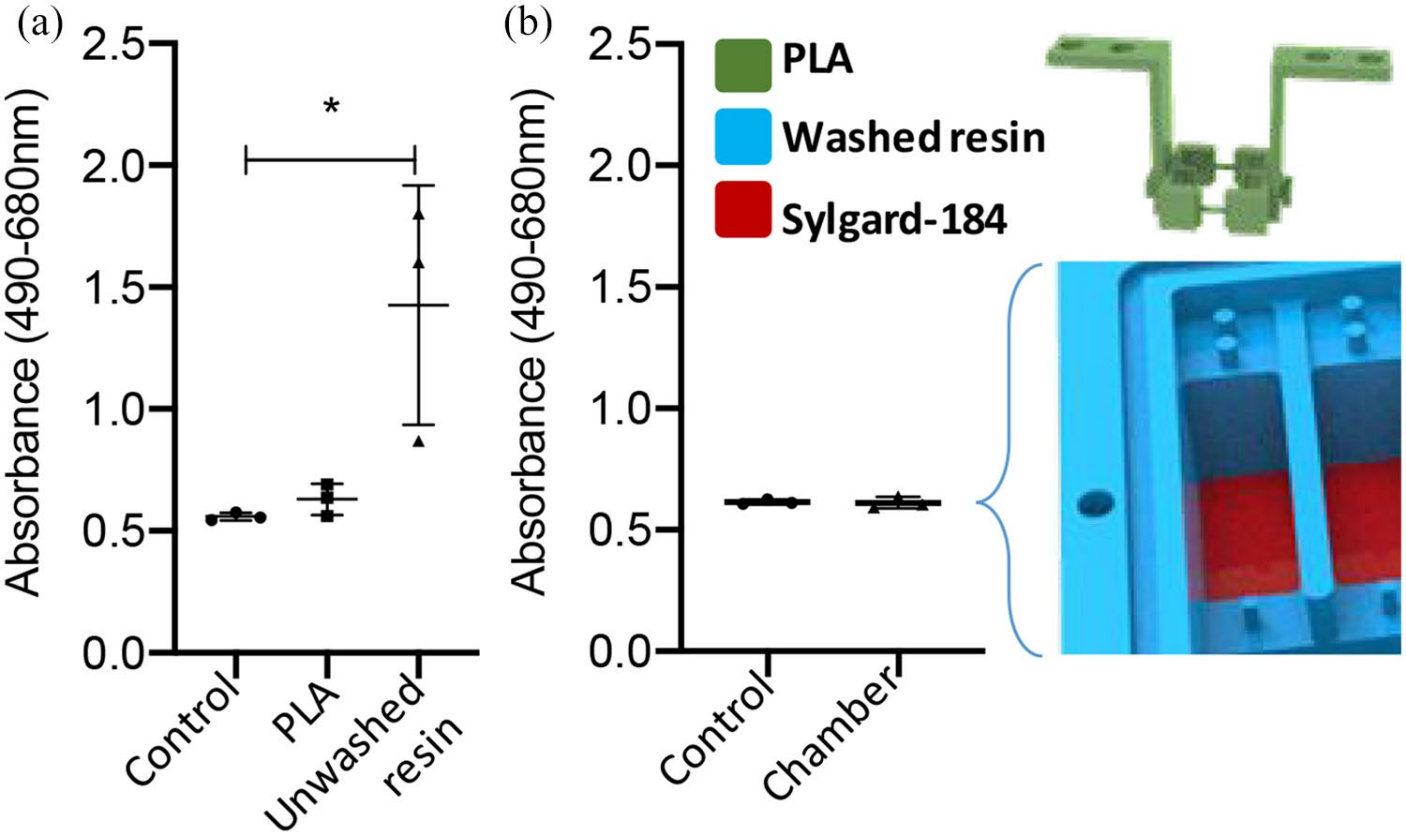
**3D printed culture chamber cytotoxicity testing.** (a) Cytotoxicity of the materials used to construct the bioreactor was tested using the LDH assay, showing no toxicity for PLA compared to tissue culture plastic controls, but significant toxicity for the freshly cured (unwashed) XTC-3D oxirane epoxy resin used to waterproof the chamber. (b) Following repeat washes in PBS to remove solvent and residual monomer, the complete culture chamber coated in XTC oxirane resin and with Sylgard-184 coated well bases was shown to have no cytotoxicity (n = 3, t-test * indicates *p* *<* 0.05). Error bars represent standard deviation.

Biocompatibility and cytotoxicity were evaluated using the LDH assay, demonstrating that PLA had no cytotoxicity and was equivalent to tissue culture plastic controls (Figure 4). The XTC-3D resin was initially found to induce significantly more LDH release from hMSCs (2.5-fold increase, *p* < 0.05), indicating some cytotoxicity (Figure 4(a)). The LDH assay was performed again, this time in the fully coated, cured and assembled bioreactor chamber. As described above, the culture wells were coated in XTC-3D oxirane resin and the base was coated in Sylgard-184. After the resins had completely cured, the culture wells were thoroughly washed in PBS and the LDH assay was repeated within the bioreactor chamber, yielding equivalent LDH activity values to controls (six-well plate) (Figure 4(b)). These data confirm that repeated washing renders XTC-3D oxirane resin and Sylgard-184 biocompatible by removal of any residual toxic monomers.

**Figure 5.**
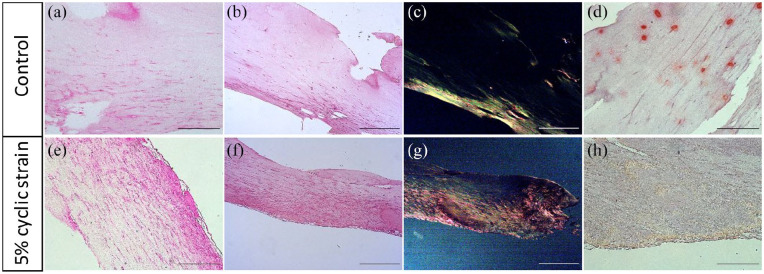
**Histology sections of tissue-engineered tendons following 21**-**day periodic cyclic strain.** Tissue-engineered tendons received 5% strain at 1 Hz for 5 h/day and were compared to un-stretched controls. Representative images are shown for haematoxylin and eosin to show cell bodies and nuclei (a and e), Picrosirius Red to show collagen deposition under regular transmission (b and f) and polarised light microscopy (c and g) and Alizarin Red staining for calcification (d and h). Under 5% cyclic strain, tendons showed increased alignment of cells and collagenous matrix, and avoided calcification in culture. Scale bar represents 0.5 mm.

Histology was used to determine structural changes in the tissue-engineered tendons in response to applied strain (Figure 5). H&E staining suggested that under 5% dynamic strain, tendons produced a more aligned fibrous matrix than controls cultured under static tension (Figure 5(a) and (E)). Picrosirius red was used to stain collagen, with stain intensity under brightfield illumination highlighting areas of increased collagen deposition, most notably at the outer surface of the tendons cultured with the dynamic strain (Figure 5(b) and (f)). Under polarised light, the picrosirius-stained collagen appeared as red, green and yellow showing progressively increased alignment of the fibres with dynamic tensile stimulation (Figure 5(c) and (g)). Alizarin red was used to determine calcification or mineralisation of the collagenous matrix. Under static strain, the control tendons accumulated multiple focal calcium deposits which did not appear to be present in any of the dynamically stretched tendons (Figure 5(d) and (h)).

**Figure 6.**
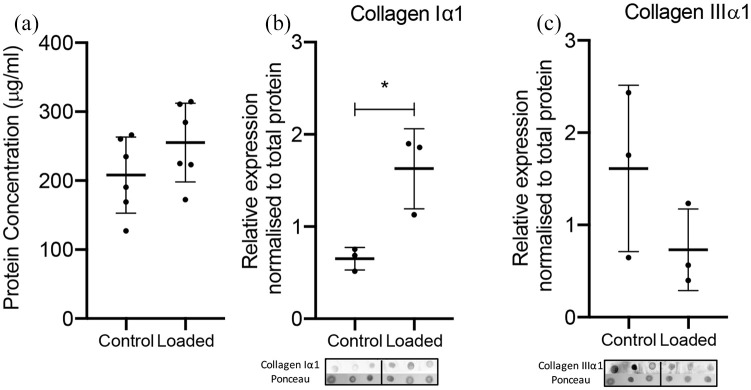
**Collagen production by cells in the 3D printed culture chamber**. (a) BCA assay shows protein content for all wells. n = 6 repeats. Error bars represent standard deviation. Significance measured using Student’s t-test. Densitometric comparison and corresponding dot blot images of Collagen Iα1 (b) and Collagen IIIα1 (c) expression in control and 5% cyclically strained samples. n = 3 repeats. Error bars represent standard deviation. Significance measured by Student’s t-test. * *p* *<* 0.05.

BCA assay results of six samples from both control and loaded conditions showed the protein concentration was similarly normally distributed around the mean across all wells in both control and stretched groups (Figure 6(a)). Semi-quantitative analysis of collagen I and III depositions in the tendon was performed by dot blot assay after 35 days total culture: 14 days contraction in well plates followed by 21 days in the bioreactors (Figure 5). Cyclic strain resulted in a significant 2.5-fold increase in collagen Iα1 content compared to controls (*p* = 0.0197), and reduced collagen IIIα1 content (*p* = 0.1973).

## Discussion

In this investigation, we have shown that 3D printing is a useful method for producing customisable bioreactors, in this case, to overcome technological challenges in tendon tissue bioengineering. Our objectives were to produce a low-cost and easily replicable six-well bioreactor chamber that could be broadly adopted by the tissue engineering community using existing bioreactors and base actuator platforms, thereby improving the consistency and availability of research tools. The culture chamber we have developed is directly comparable in function and performance to existing commercial systems, and is adaptable to multiple base-platforms (e.g. those manufactured by EBERS and CellScale) enabling electronics, motor assemblies and software to be used. We developed our culture chamber design using the EBERS TC-3 bioreactor platform as a base, keeping the linear motor assembly and proprietary software to drive the actuator arm. Design of the original prototypes through to final product validation took approximately 7 months and serial redesigns were used to enhance the specification and optimise print quality and speed. Final product validation was established using a number of tests derived from the literature, ISO specifications (e.g. ISO 10993 Cytotoxicity test for biocompatibility) and to meet the scientific requirements for our subsequent research in tendon bioengineering.

Tests were performed to determine the accuracy of the 3D print, and validate that the actual displacement across each of the six bioreactor wells matched the programmed value. Displacement was initially measured using a flexible resistance wire technique described in Banik and Brown,^39^ but this was found to produce highly variable values in our investigation. Displacement was instead measured from 0.4 to 4 mm using a USB camera fixed above the chamber and recorded as a percentage of the total tissue-engineered tendon length (8 mm), with the distance from the wall to tensile arm measured frame by frame from video capture. Using this method, we found that displacement was uniform across each well and matched the programmed strain from 5 to 50% (±0.6 %). This correlation between applied versus recorded displacement was determined to be within acceptable engineering design limits, and is comparable to other published studies (e.g. a biaxial loading bioreactor designed by Yossuf *et al.*^46^ had a correlation of ±0.95%).

The biocompatibility and cytotoxicity of the materials used to form the chamber were assessed by the LDH assay using MSCs cultured in well plates over 24 h. The PLA base material was found to be non-toxic (no more LDH activity compared to tissue culture plastic controls), while the XTC-3D resin (oxirane epoxy) used to waterproof the 3D printed culture chamber was initially discovered to be toxic. We proposed that this toxicity resulted from the residual solvent and monomer leaching into the culture media, which could be eliminated by repeat washing in PBS (six washes), after which the fully washed resin-coated PLA showed no toxicity (LDH activity equal to tissue culture plastic controls). Other researchers have also shown that epoxy is compatible with cell culture.^47^ Our final coating, Sylgard-184, was also found to be non-toxic. Sylgard-184 is a widely used material for cell culture applications which provided a hydrophobic barrier, ensuring low protein adhesion and smooth operation of the bioreactor.

PLA is a versatile polymer and the most common material available for FFF desktop 3D printing, followed by acrylonitrile butadiene styrene (ABS) and nylon. ABS is not recommended for manufacturing the 3D printed culture chamber as it has no material advantages to PLA in this application and needs to be used in a controlled environment (e.g. a class I fume hood) due to the production of toxic fumes and particulates during high-temperature extrusion.^48,49^ Prototypes of our culture chamber were also printed in Nylon, but in our usage, the Nylon culture chamber was noticeably softer than the PLA culture chamber, resulting in difficulties in securing fixing screws which prevented the lid from correctly fitting in place. Nylon is anecdotally discussed in the online 3D printing community as useful in some biomedical applications due to its ability to withstand sterilisation by autoclave, but in our experience, parts were found to swell and distort upon exposure to aqueous media and even ambient room humidity during printing, a phenomenon reported by other authors to be caused by delamination between the deposition layers.^50^ This distortion prevented usable parts (especially large or straight parts such as the tensile displacement arm) from being fabricated in nylon. Coating non-toxic PLA in an impermeable non-toxic XTC-3D resin and Sylgard-184 was shown to be a suitable solution to manufacturing, durability and sterility requirements.^51^

Our overall objectives were to develop a bioreactor able to drive biological adaptations in cells to dynamic mechanical environments, resulting in functional changes in ECM production. To investigate these effects using this bioreactor, we cultured hMSCs in fibrin hydrogels for 21 days with the anchor points either fixed in place (controls) or cyclically stretched at 5% for 5 h/day (0.5 Hz for 5 days/week). A 5% magnitude uniaxial cyclic strain was chosen as this corresponds to accepted physiological levels of strain in the tendon,^52^ while 0.5 Hz was the practical upper limit of speed and displacement for the EBERS-TC3 linear motor driving the bioreactor arm. Howard et al.^53^ found that periodontal ligament fibroblasts exposed to 5% biaxial strain at 0.5 Hz increased collagen type I and fibronectin synthesis, and these findings are generally consistent in the literature.^22^ The duration of cyclic tensile loading varies substantially between published research articles, with short periods of 1 h^45^ to long periods up to 24 h^50,53–55^ and loading delivered at intervals or in variable regimes, for example, 6 h at 10% followed by 3% for 18 h.^18^ Morita et al.^12^ saw an increase of collagen type I gene expression with 5% tensile stimulation and investigated the response of genes associated with tenogensis and tendon ECM: tenascin C, scleraxis, collagen I and collagen III. Scleraxis was quickly upregulated being significantly more highly expressed after 24 h over unloaded controls, while tenascin, collagen I and collagen III were all significantly increased after 48 h, data which are supported by several other studies in response to 5% tensile strain.^22,56^

We used conventional histological techniques to determine changes in cell alignment and protein production. H&E staining revealed that the tissue-engineered tendon cultured with dynamic 5% strain had a more visibly pronounced alignment and a greater abundance of cells and matrix at the surface than controls, supporting findings from previous studies.^10,57^ Picrosirius staining was used to further characterise the increased concentration and alignment of collagen fibres produced by cells under dynamic 5% strain. Alizarin red staining was used to detect mineralisation, as the tissue-engineered tendon cultured in FCS-supplemented DMEM has been shown by some authors to result in calcification atypical of healthy native tendon.^58^ We confirmed that there was evidence of calcification in fibrin tendon construct grown under static strain which was not present when tissue-engineered tendons were dynamically stretched.

Variation in protein synthesis across the bioreactor chamber was low, and similar under both control and strained conditions, further indicating consistency in the loading across all six wells of the bioreactor chamber. To assess differences in collagen biosynthesis in response to either static or dynamic strain, a dot blot analysis of the tissue-engineered tendon was performed after 21 days. Collagen Iα1 production was found to be 2.5-fold higher in the dynamic samples compared to controls, while collagen IIIα1 production appeared slightly suppressed but not significantly different between static or dynamic strain. Variability in the control group production of collagen IIIα1 was high, however (standard deviation, 0.9, n = 3), compared to the loaded group (standard deviation, 0.4, n = 3). Taken together, these results show evidence of differential transcriptional responses and ECM adaptation in response to either static or dynamic strain which supports our continued investigation using more advanced and quantifiable approaches.

## Conclusion

Using sharable CAD and 3D printing, we have designed, manufactured and tested a culture chamber that enables equal tensile forces to be applied to six isolated, independent samples in one chamber over a large (0–4 mm) displacement range. The materials used in the design are readily available for desktop manufacturing, easy to work with and were shown to be durable and non-toxic once the appropriate curing, washing and processing techniques had been applied. The bioreactor chamber was successfully sterilised with 70% ethanol and was used for 18 months without infection, highlighting its reusability.

The bioreactor performed as designed in delivering mechanical strain to tissue-engineered tendons. Tissue-engineered tendon containing hMSCs cultured under periodic cyclic strain exhibited changes in structural alignment and protein production, with a significant increase in collagen Iα1 (the most abundant polypeptide in tendon) and the absence of matrix calcification consistent with tenogenic differentiation.

The design and optimisation of this bioreactor provide a freely available and globally reproducible platform for ongoing comparative research in tendon biology and tissue engineering. The ease and speed of the 3D printing process allow for multiple 3D printed chambers to be manufactured for each experiment ensuring the control chamber is identical to the experimental chambers, which is often a compromise with high-cost commercially available bioreactor systems where the number and availability of culture chambers are often limited to a single unit.^11,34^ With minimal edits to the design of the 3D printed culture chamber baseplate, the system can be mounted onto a range of commercially available and custom-made actuators.
